# Body Composition Attenuates the Association Between Serum 25-Hydroxyvitamin D and Bone Mineral Density in Early Postmenopausal Women

**DOI:** 10.3390/nu18050865

**Published:** 2026-03-07

**Authors:** Raquel Domingo-Molina, Borja Sañudo, Sergio Tejero, Gonzalo Reverte-Pagola, Mª Ángeles Martínez-Maestre

**Affiliations:** 1 Department of Obstetrics and Gynecology, Hospital Universitario Virgen del Rocío, Biomedicine Institute of Seville (IBIS), CSIC, 41013 Seville, Spain; 2 Department of Physical Education and Sport, University of Seville, 41013 Seville, Spain; 3 Foot and Ankle Unit, Orthopaedic Department, Department of Surgery, University Hospital Virgen del Rocío, University of Seville, 41013 Seville, Spain; tejerogarciasergio@gmail.com; 4 School of Humanities, Education and Sport, Universidad CEU Fernando III, 41930 Seville, Spain

**Keywords:** vitamin D, 25-hydroxyvitamin D, bone mineral density, body composition, lean mass, postmenopause, dual-energy X-ray absorptiometry, osteoporosis prevention

## Abstract

**Background/Objectives**: Vitamin D plays a central role in calcium and bone homeostasis; however, evidence linking serum 25-hydroxyvitamin D (25(OH)D) to bone mineral density (BMD) in postmenopausal women remains inconsistent. Because body weight and lean mass strongly influence skeletal loading and may also affect circulating 25(OH)D, we aimed to evaluate the association between serum 25(OH)D and bone outcomes in early postmenopausal women and to determine whether body composition attenuates this relationship. **Methods**: In this cross-sectional study, 120 women within 10 years after natural menopause (59.5 ± 6.3 years) were assessed. Serum 25(OH)D was measured by chemiluminescent immunoassay. Total body areal bone mineral density (total body aBMD, g/cm^2^) was assessed by DXA, and trabecular volumetric BMD and cortical thickness were obtained using 3D modeling. Associations were examined using Spearman correlations and multivariable linear and logistic regression models adjusted for age, body weight, lean mass, and years since menopause. **Results**: Median serum 25(OH)D was 23.7 ng/mL [16.7–30.4]. A modest correlation was observed between 25(OH)D and total body aBMD (ρ = 0.22, *p* = 0.016), but not with trabecular volumetric BMD or cortical thickness. After adjustment, 25(OH)D was not independently associated with total body aBMD (*p* = 0.144), whereas body weight remained significantly associated (β = 0.27, *p* = 0.002). In logistic models, body weight (OR = 0.93, 95% CI 0.90–0.96) and lean mass (OR = 0.97, 95% CI 0.95–0.99) were protective against low BMD, while the association with 25(OH)D was modest. **Conclusions**: In early postmenopause, the association between serum 25(OH)D and BMD is modest and largely attenuated after accounting for body composition. Body weight and lean mass appear to be stronger determinants of bone outcomes than vitamin D status.

## 1. Introduction

Vitamin D is central to mineral and bone homeostasis. Its active metabolite, 1,25-dihydroxyvitamin D, promotes intestinal absorption of calcium and phosphate and modulates bone remodeling by acting on osteoblasts and osteoclasts via the vitamin D receptor. Consequently, circulating 25-hydroxyvitamin D (25(OH)D) is widely accepted as the most reliable biomarker of vitamin D status because it integrates multiple determinants, including cutaneous synthesis from sunlight exposure, dietary intake, supplementation, intestinal absorption, and sequestration in adipose tissue [1]. Consequently, serum 25(OH)D reflects total vitamin D availability at the systemic level more accurately than dietary intake alone. This distinction is particularly relevant in Mediterranean populations, where habitual dietary vitamin D intake is typically low and relatively homogeneous, and interindividual variability in 25(OH)D concentrations is largely driven by effective sun exposure, body composition, and supplementation patterns [2].

For this reason, examining circulating 25(OH)D is widely considered a biomarker of vitamin D status with potential implications for bone mineral density (BMD) and fracture risk, as it provides a physiologically integrated measure of vitamin D availability relevant to skeletal outcomes [3,4,5]. Nevertheless, epidemiological and interventional data relating 25(OH)D to BMD are inconsistent [4,5]. While some observational studies report positive associations, randomized supplementation trials often fail to demonstrate meaningful BMD benefits except in markedly deficient individuals [6,7], suggesting that the skeletal effects of vitamin D may depend on contextual factors beyond circulating 25(OH)D alone [2,8,9].

This uncertainty is particularly relevant in postmenopausal women, among whom the abrupt fall in estrogen precipitates a phase of accelerated bone turnover and rapid BMD loss [6]. Estrogen deficiency is also accompanied by a state of chronic low-grade inflammation, characterized by increased production of pro-inflammatory cytokines that promote osteoclastogenesis and impair osteoblast function [7]. This inflammatory milieu contributes to accelerated bone loss in postmenopausal women and may interact with endocrine and metabolic factors, including vitamin D metabolism and body composition [8]. Early postmenopause, commonly defined as the first decade after the final menstrual period, is characterized by disproportionately large decreases in trabecular bone and deterioration of microarchitecture, processes that substantially contribute to long-term fracture risk [9]. While adequate vitamin D status supports calcium balance and bone remodeling, mounting evidence indicates that anthropometry and body composition exert strong influences on BMD in this population. This effect may be particularly relevant during early postmenopause, a period characterized by rapid hormonal changes and accelerated bone loss [10,11,12]. From a nutritional perspective, adequate dietary protein intake supports bone health primarily through its effects on muscle mass, IGF-1 signaling, and bone remodeling. Given the close functional coupling between muscle and bone, nutritional influences on skeletal integrity may therefore operate indirectly via lean mass and mechanical loading [13]. Despite this critical phase of accelerated skeletal loss, early postmenopause remains underrepresented in studies specifically addressing determinants of bone density.

Body composition may confound or mediate the observed associations between 25(OH)D and bone [1,14]. Adipose tissue acts as a reservoir for lipophilic vitamin D metabolites, potentially lowering circulating 25(OH)D through sequestration, whereas higher body weight and lean mass enhance skeletal loading and promote bone formation through mechanical and endocrine mechanisms; both pathways are biologically plausible and empirically supported [1].

Recent population studies suggest that when weight and lean mass are accounted for, the direct association between 25(OH)D and BMD is attenuated, implying that part of the apparent vitamin D–bone link may be driven by body composition rather than by 25(OH)D per se [14,15].

Despite extensive research on vitamin D and skeletal health across heterogeneous postmenopausal samples, relatively few studies have targeted early postmenopause as a distinct window of vulnerability [16,17,18]. Most studies include heterogeneous postmenopausal samples or focus on late postmenopause, potentially obscuring changes specific to the early transition when bone loss is most rapid [19,20,21]. In particular, the joint contribution of serum 25(OH)D and body composition to trabecular volumetric BMD and cortical structure has not been adequately characterized in early postmenopausal women. Accordingly, this study aimed to evaluate the association between serum 25(OH)D and detailed bone outcomes (total areal BMD, trabecular volumetric BMD and cortical thickness) in women within the first ten years after menopause, and to assess whether body composition (specifically body weight and lean mass) influences or accounts for variation in this relationship. By restricting the sample to early postmenopause and combining volumetric and cortical assessments with body composition variables, we provide novel evidence on whether 25(OH)D is independently associated with bone or whether observed associations are primarily driven by body composition, information with direct implications for targeted prevention of bone loss during a clinically critical period.

## 2. Materials and Methods

### 2.1. Study Design and Participants

This analysis is a cross-sectional study utilizing baseline data collected from the WEAPOM Project, a registered randomized controlled trial (ClinicalTrials.gov identifier: NCT06741956) primarily designed to evaluate the efficacy of an mHealth-based impact exercise intervention for bone health enhancement in postmenopausal women [22]. Only pre-intervention data were used, and no participant had initiated any component of the intervention at the time of assessment. The study was approved by the Andalusian Biomedical Research Ethics Committee (approval code: 0486-N-22), and all participants provided written informed consent in accordance with the Declaration of Helsinki.

A total of 120 postmenopausal women within ten years after natural menopause (mean ± SD age: 59.5 ± 6.3 years) were included. Participants were recruited from gynecology units at the Virgen del Rocío University Hospital (Seville, Spain), through local media advertisements, social networks, and information brochures distributed in primary care centers. Participant recruitment and baseline data collection were conducted between February 2025 and May 2025. All assessments were performed during this period prior to initiation of the intervention. The duration of data collection was approximately three months. Because serum 25(OH)D concentrations may exhibit seasonal variability, the date of blood sampling was recorded. However, the study was not specifically designed to evaluate seasonal effects, and season was not included as a covariate in the statistical models. Baseline assessments were conducted within a restricted time window (February to May 2025), thereby limiting potential seasonal variability within the cohort.

Inclusion criteria were age over 40 years old and within 10 years of menopause onset, time since last menstrual period ≥ 12 months, and body mass index (BMI) between 18 and 35 kg/m^2^. Postmenopausal status was defined according to established international consensus criteria as the absence of menstruation for at least 12 consecutive months in the absence of other physiological or pathological causes [6]. In women within the typical age range, routine hormonal measurements (e.g., FSH or estradiol) are not required for diagnosis, as clinical criteria are considered sufficient.

Eligible participants must be physically inactive, defined as engaging in less than 150 min of moderate to vigorous physical activity per week over the past 6 months, and must provide written informed consent. Exclusion criteria included surgically induced menopause, active cancer, alcohol intake > 14 standard drinks/week, current smoking, use of hormone therapy within the past three months, recent fracture (<6 months), diagnosis of osteoporosis, severe balance disorders, or mobility limitations that could preclude assessment or physical activity participation. Eligibility criteria were verified through structured clinical interviews, review of electronic medical records, and objective clinical assessments conducted at the study center. Menopausal status was corroborated during gynecological evaluation. Clinical exclusion criteria, including prior diagnosis of osteoporosis, recent fractures, malignancy, and hormone therapy use, were confirmed through review of hospital medical records. Lifestyle-related variables, such as smoking status, alcohol intake, and physical activity level, were collected using standardized questionnaires administered by trained research personnel. No inclusion or exclusion decisions were based solely on unverified self-reports.

All assessments were conducted at the Duque del Infantado Specialty Center (Virgen del Rocío University Hospital, Seville). Assessments were comprehensive and included clinical history, blood sampling, physical fitness tests, Dual-Energy X-ray Absorptiometry (DXA), and advanced 3D structural bone analysis:

#### 2.1.1. Serum 25-Hydroxyvitamin D Concentration

Venous blood samples were collected during the baseline assessment to determine serum 25(OH)D concentrations using chemiluminescent immunoassay (Liaison^®^, DiaSorin, Italy). Results were expressed in ng/mL and categorized as deficient (<20 ng/mL), insufficient (20–30 ng/mL), or sufficient (>30 ng/mL), following Endocrine Society guidelines [23].

#### 2.1.2. Areal Bone Mineral Density and Body Composition by DXA

Areal BMD (aBMD, g/cm^2^) was measured using DXA (Hologic Discovery Wi^®^, Hologic Inc., Marlborough, MA, USA) at the lumbar spine (L1–L4) and proximal femur (femoral neck and total hip). Certified technicians performed all scans, and daily calibration and quality control procedures were implemented according to manufacturer specifications. In addition, whole-body DXA scans were performed in all participants, from which total body areal bone mineral density (total body aBMD, g/cm^2^) was derived. Total body aBMD was selected as the primary dependent variable for the main regression analyses in the present cross-sectional study. Whole-body composition was simultaneously assessed by DXA, providing measurements for total lean mass (g), total fat mass (g), and Body Mass Index (kg/m^2^). These body composition metrics were included as covariates in subsequent analyses to evaluate their influence on the observed associations between vitamin D status and bone health.

#### 2.1.3. Three-Dimensional Bone Structural Analysis (3D-Shaper)

To derive detailed insights into bone geometry and microarchitecture, the DXA scans of the proximal femur were post-processed using the 3D Shaper^®^ software (v2.14.0, Galgo Medical, Barcelona, Spain). This three-dimensional modeling approach, validated against quantitative computed tomography (QCT), allows evaluation of bone microstructure and estimated strength [24,25] and permitted the estimation of key structural parameters, including: total and trabecular volumetric BMD (vBMD, mg/cm^3^), cortical bone density (mg/cm^3^), and cortical thickness (mm). These three-dimensional structural parameters were analyzed as secondary, exploratory outcomes.

### 2.2. Statistical Analysis

All statistical analyses were performed using Python version 3.11 (Python Software Foundation, Wilmington, DE, USA), with the pandas, scipy, numpy, and matplotlib libraries. Continuous variables were tested for normality using the Kolmogorov–Smirnov test. Normally distributed data are expressed as mean ± standard deviation (SD), and non-normally distributed variables (e.g., serum 25-hydroxyvitamin D [25(OH)D]) as median [interquartile range, IQR]. Bivariate analyses were performed using Spearman’s rank correlation coefficient (ρ) to assess associations between serum 25(OH)D and bone parameters (total body aBMD, trabecular volumetric BMD, cortical thickness), as well as with body composition variables (weight, total fat mass, total lean mass).

Multiple linear regression models were constructed to assess the independent association between serum 25(OH)D concentration and total body aBMD (g/cm^2^), adjusting for age (years), body weight (kg), lean mass (g), and years since menopause. Covariates were selected a priori based on biological plausibility and prior literature indicating their association with both vitamin D status and bone mineral density. Model assumptions were verified by examination of residuals, independence of errors (Durbin–Watson statistic), and multicollinearity (variance inflation factor < 5). A binary logistic regression model was used to evaluate predictors of low total body aBMD (≤1 SD below the sample mean). In this model, serum 25(OH)D was entered as a continuous variable (ng/mL). The multivariable logistic model was adjusted for age, body weight, lean mass, and age at menopause. This distribution-based threshold (≤1 SD below the sample mean) was used as an exploratory within-cohort cut-off to identify participants at the lower end of the BMD distribution, rather than as a clinical diagnostic criterion. Low total body aBMD was defined as values ≤ 1 SD below the sample mean to facilitate complementary interpretation alongside the primary continuous analyses, given the focus on within-sample variability and early skeletal changes. Odds ratios (ORs) and 95% confidence intervals (CIs) were estimated both crudely and after adjustment for age, body weight, and lean mass. Model fit was assessed with the Hosmer–Lemeshow goodness-of-fit test and Nagelkerke’s R^2^. Statistical significance was defined as two-tailed *p* < 0.05. 

**Figure 1:**
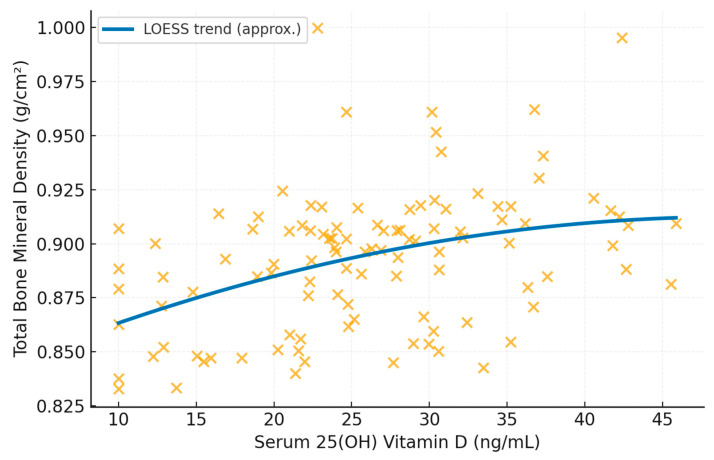
Association between Serum 25(OH)D and total body aBMD in postmenopausal women.

For multivariable regression analyses, a complete-case approach was applied. Participants with missing data in any of the variables included in the adjusted models (serum 25-hydroxyvitamin D, body weight, lean mass, age, or years since menopause) were excluded from these analyses. As a result, the final sample size for multivariable models was n = 108. For graphical representation of the association between serum 25-hydroxyvitamin D and total body aBMD (Figure 1), the same analytical sample (*n* = 108) was used to ensure consistency between visualization and regression analyses. No additional exclusions were applied beyond those related to missing data.

## 3. Results

A total of 120 postmenopausal women were included in the analysis. The mean age was 59.5 ± 6.3 years, with a median time since menopause of 8.2 years. Median serum 25(OH)D concentration was 23.7 ng/mL [IQR 16.7–30.4], indicating a wide range of vitamin D status. Mean values for bone health parameters were: total body aBMD 0.911 ± 0.101 g/cm^2^, trabecular vBMD 151.0 ± 46.2 mg/cm^3^, and cortical thickness 1.85 ± 0.15 mm (Table 1).

Bivariate analyses revealed a modest but statistically significant association between serum 25(OH)D and total body aBMD (ρ = 0.22, *p* = 0.016) (Figure 1). However, this association was not observed for trabecular volumetric vBMD (ρ = 0.11, *p* = 0.22) or cortical thickness (ρ = 0.09, *p* = 0.31) and was no longer statistically significant after adjustment for body composition variables included in multivariable models.

In the multiple linear regression model (Table 2a), the overall model was significant (F [5102] = 8.94, *p* < 0.001), explaining 28.5% of the variance in total body aBMD (adjusted R^2^ = 0.285). After adjustment for covariates, 25(OH)D was not an independent predictor of total body aBMD (β = 0.125; *p* = 0.144). Body weight was the only variable that remained significantly associated with total body aBMD (β = 0.270; *p* = 0.002), indicating that each 1 kg increase in weight was associated with an approximate increase of 0.002 g/cm^2^ in total body aBMD. Age, lean mass, and years since menopause were not statistically significant predictors (all *p* > 0.10). Multicollinearity diagnostics indicated low variance inflation factors for all predictors (25(OH)D = 1.06; body weight = 2.99; lean mass = 2.97; age = 2.18; years since menopause = 2.15), indicating no evidence of problematic collinearity.

In the logistic regression model predicting low BMD (<0.85 g/cm^2^; Table 2b), defined as total body aBMD values ≤ 1 SD below the sample mean, serum 25(OH)D was modeled as a continuous predictor (ng/mL). The multivariable model was adjusted for age, body weight, lean mass, and age at menopause. The overall model fit was satisfactory (Nagelkerke R^2^ = 0.36; Hosmer–Lemeshow *p* = 0.42), with no evidence of multicollinearity (VIF < 2 for all covariates). Body weight demonstrated the strongest protective association (OR = 0.93, 95% CI 0.90–0.96, *p* < 0.001), followed by lean mass (OR = 0.97, 95% CI 0.95–0.99, *p* = 0.005). Conversely, age increased the odds of low BMD by 4% per year (OR = 1.04, 95% CI 1.01–1.07, *p* = 0.002), while later age at menopause showed a nonsignificant trend toward protection (OR = 0.98, 95% CI 0.95–1.00, *p* = 0.075). This categorical analysis was performed as a secondary, complementary approach to aid interpretation of relative associations at the lower end of the BMD distribution, while continuous total body aBMD remained the primary outcome of interest.

In the multivariable logistic regression model lower serum 25(OH)D levels were associated with a greater likelihood of low BMD (OR = 0.96, 95% CI 0.93–0.99, *p* = 0.020); however, the magnitude of this association was modest compared with that observed for body weight and lean mass, and findings should be interpreted in the context of the exploratory, distribution-based definition of low BMD.

## 4. Discussion

In this cohort of early postmenopausal women, serum 25(OH)D concentrations showed a modest positive correlation with total body aBMD, consistent with the proposed beneficial role of vitamin D in skeletal maintenance. However, this association was no longer significant after adjustment for key covariates, particularly body weight and lean mass, suggesting that adjustment for body composition substantially attenuates the observed association between vitamin D status and bone density, rather than supporting an independent effect of circulating 25(OH)D. Among the variables examined, body weight emerged as the only independent predictor of total body aBMD in the linear regression model and the strongest predictor in the logistic regression, indicating that mechanical loading and mass-related factors outweigh the direct biochemical influence of circulating 25(OH)D on bone mass. While linear models evaluate associations across the full continuous distribution of total body aBMD, the logistic model focuses on a distribution-based threshold identifying individuals at the lower end of the BMD spectrum. It should be emphasized that the logistic regression analysis was based on a within-sample, distribution-based definition of low BMD rather than established clinical cut-points, and was included as a secondary, exploratory approach complementary to the primary analyses based on continuous total body aBMD.

These findings highlight the dominant contribution of anthropometric and compositional characteristics to bone health during the early postmenopausal phase, a critical period of accelerated bone loss driven by estrogen deficiency [9,10,11]. Collectively, the results are consistent with the notion that vitamin D–bone associations observed in population studies may, in part, reflect differences in body composition rather than independent endocrine effects of vitamin D per se [1,14].

Although vitamin D plays an undisputed role in calcium absorption and bone remodeling [4,5,26], evidence from large observational and interventional studies remains inconsistent, with little impact on BMD except under conditions of severe deficiency [27,28]. Our results align with this literature, suggesting that within physiologic ranges, 25(OH)D exerts a permissive rather than a determinant role in bone mass regulation. Body composition exerts both mechanical and endocrine influences on bone strength [14]. Higher body weight enhances skeletal loading and osteoblastic activity through mechano-transduction, while lean mass contributes via muscle-derived signaling molecules such as irisin and osteocalcin [10,11,29]. Conversely, adipose tissue acts as a reservoir for vitamin D metabolites, reducing circulating 25(OH)D without necessarily impairing bioavailability or bone outcomes [1]. Consistent with prior reports, our findings indicate that once weight or lean mass is considered, the apparent vitamin D–BMD association is markedly attenuated, supporting a substantial attenuation of the vitamin D–bone association after accounting for body composition [30,31,32].

Focusing on early postmenopause provides a critical physiological context for interpreting these findings. This period is characterized by accelerated loss of trabecular bone, thinning of cortical structures, and deterioration of bone microarchitecture, changes that collectively drive the steep decline in bone strength and increased fracture risk observed during this phase [9,33,34,35]. The rapid reduction in circulating estrogens alters not only bone remodeling dynamics but also systemic metabolism, potentially affecting vitamin D metabolism, calcium handling, and the distribution of body fat and lean tissue [5,9,12,14]. Estrogen deficiency has been shown to reduce the efficiency of intestinal calcium absorption and to modify hepatic hydroxylation of vitamin D metabolites, thereby influencing their circulating levels [26,36,37]. Concurrently, the redistribution of body composition commonly seen after menopause, characterized by increased adiposity and decreased lean mass, may further modulate both 25(OH)D bioavailability and skeletal loading forces [12]. In this transitional context, the observed protective role of body weight in our study may reflect a compensatory influence that partially offsets the deleterious effects of estrogen withdrawal on bone. Mechanical loading associated with higher weight, as well as endocrine factors derived from muscle and adipose tissue, could help maintain bone turnover balance and mitigate trabecular loss during early postmenopause [10,11]. These findings underscore the importance of maintaining adequate body mass and muscle strength in the early postmenopausal years, not only for metabolic health but also as a key determinant of skeletal integrity during a period of heightened vulnerability. Taken together, these biological considerations may help explain why the skeletal actions of vitamin D are not exclusively dependent on its serum concentration but are instead integrated within a broader physiological context influenced by body composition, mechanical loading, and metabolic signaling. Accordingly, the relationship between vitamin D and bone health in postmenopausal women may vary according to individual differences in body composition and mechanical loading, reflecting the interplay between vitamin D metabolism and the biomechanical and endocrine characteristics of the individual.

Clinically, these findings suggest that while serum 25(OH)D remains an important marker of bone metabolism, assessment of bone health should also consider body composition, particularly body weight and muscle mass, as key determinants of skeletal integrity. Maintaining adequate lean mass and healthy weight through lifestyle interventions focused on mechanical loading and nutritional optimization may provide synergistic skeletal benefits beyond vitamin D supplementation [12]. Supplementation remains warranted for women with marked deficiency or low body weight, where both metabolic and mechanical support to bone are reduced [27,28]. Overall, this study supports an integrated clinical approach in which vitamin D optimization is coupled with strategies aimed at improving or maintaining body composition. Such a multidimensional model, addressing endocrine, nutritional, and mechanical determinants, may be particularly effective in mitigating the rapid bone loss characteristic of early postmenopause and in reducing long-term fracture risk at the population level.

A key strength of this study lies in the homogeneity of the sample, composed exclusively of women within the first decade after menopause, a critical yet often understudied phase of accelerated skeletal remodeling. This focused design minimizes confounding by menopausal duration and allows for a clearer interpretation of vitamin D–bone relationships specific to early postmenopause. Another notable advantage is the comprehensive assessment of bone parameters, including total body aBMD, trabecular volumetric BMD, and cortical thickness, providing a multidimensional evaluation of bone health beyond conventional densitometry. Furthermore, the application of multivariable regression models enabled adjustment for relevant covariates and facilitated evaluation of how accounting for body composition influences observed vitamin D–bone associations, contributing to a more nuanced interpretation of the findings. However, several limitations should be acknowledged.

A key limitation is the potential for residual confounding, which is a well-recognized challenge in observational vitamin D research. Although analyses were adjusted for major anthropometric determinants of bone health, several factors known to influence circulating 25-hydroxyvitamin D concentrations were not systematically collected and therefore could not be included in the adjustment set. These include season or timing of blood sampling, detailed information on vitamin D supplementation, habitual sunlight exposure, and dietary intake of vitamin D and calcium. In addition, relevant biochemical markers that could provide further physiological context, such as parathyroid hormone levels or renal function, were not available. While circulating 25(OH)D was selected as the exposure of interest because it represents an integrated biomarker reflecting the combined effects of sunlight exposure, dietary intake, supplementation, absorption, and adipose tissue sequestration, unmeasured upstream determinants may still have influenced the observed associations. Consequently, the findings should be interpreted as adjusted associations rather than evidence of independent or causal effects of vitamin D on bone outcomes.

First, the cross-sectional design precludes causal inference. Longitudinal studies are required to determine temporal relationships between 25(OH)D, body composition, and bone changes over time. Second, dietary intake was not directly assessed, including vitamin D, calcium, and protein consumption, nor was detailed information on vitamin D supplementation systematically collected. As adequate protein intake is known to influence bone health indirectly through its effects on muscle mass, IGF-1 signaling, and bone remodeling, the absence of dietary data limits our ability to fully characterize the nutritional pathways that may contribute to the observed associations, particularly those mediated by lean mass [13]. Third, inflammatory biomarkers were not measured, precluding evaluation of the potential contribution of chronic low-grade inflammation to bone loss in this cohort. Given that estrogen deficiency in postmenopausal women is associated with increased inflammatory activity that promotes bone resorption and impairs bone formation, unmeasured inflammatory status may have influenced bone outcomes independently of vitamin D and body composition [8]. Although blood sampling occurred within a restricted time frame (February to May 2025), season was not formally operationalized or included as a covariate in the statistical models. Therefore, residual confounding related to seasonal variation in serum 25(OH)D cannot be entirely excluded. Finally, the moderate sample size (*n* = 120) may have limited statistical power to detect small but clinically relevant effects, particularly for trabecular or cortical parameters. Lastly, analyses examining attenuation after covariate adjustment were exploratory and based on regression-based inference rather than formal mediation or causal modeling and should therefore be interpreted as hypothesis-generating.

## 5. Conclusions

Despite these constraints, the study provides valuable and internally consistent evidence that advances understanding of the interplay between vitamin D status, body composition, and bone health during the early postmenopausal period. In early postmenopausal women, the association between serum 25(OH)D and total body aBMD is substantially attenuated after adjustment for body composition, providing evidence against a strong independent direct skeletal effect of vitamin D.

Body weight and lean mass emerged as the principal determinants of bone density and strength, indicating that mechanical and metabolic influences associated with body composition are dominant contributors during this phase of accelerated bone loss. These findings emphasize the need for an integrated preventive strategy that combines optimization of vitamin D status with the preservation of muscle mass and adequate body weight. Such an approach may be particularly relevant to mitigate early postmenopausal bone deterioration and support long-term skeletal health.

## Figures and Tables

**Table 1:** Baseline characteristics of early postmenopausal women included in the study.

Variable	Value
Age (years)	* 59.00 [54.0–64.0]
Weight (kg)	* 68.50 [61.2–75.6]
Serum 25-hydroxyvitamin D (25(OH)D) (ng/mL)	* 23.70 [16.7–30.4]
Total body areal BMD (aBMD, g/cm^2^)	0.911 ± 0.101
Trabecular vBMD (mg/cm^3^)	151.0 ± 46.2
Cortical Thickness (mm)	1.85 ± 0.15

Values are expressed as median [interquartile range] (*) or mean ± standard deviation.

**Table 2:** (**a**) Multiple linear regression model evaluating predictors of total body aBMD in postmenopausal women. (**b**) Multivariable logistic regression analysis of predictors of low bone mineral density in early postmenopausal women.

(**a**)						
**Predictor**	**B (Unstandardized)**	**SE**	**β (Standardized)**	**95% CI for B**	***p* Value**	
Serum 25-hydroxyvitamin D (25(OH)D), ng/mL	0.0012	0.0008	0.125	[−0.0004; 0.0028]	0.144	
Weight (kg)	0.0020	0.0006	0.270	[0.0008; 0.0032]	0.002 *	
Lean mass (kg)	0.003	0.002	0.108	[−0.001; 0.007]	0.158	
Age (years)	−0.0015	0.0010	−0.095	[−0.0034; 0.0004]	0.123	
Years since menopause	0.0012	0.0009	0.086	[−0.0006; 0.0030]	0.194	
Constant	0.721	0.082	—	[0.559; 0.883]	<0.001 *	
* Values represent unstandardized (B) and standardized (β) regression coefficients with standard errors (SE) and 95% confidence intervals (CI). The dependent variable is total body areal bone mineral density (aBMD, g/cm^2^). Model adjusted for serum 25-hydroxyvitamin D [25(OH)D], body weight, lean mass, age, and years since menopause. Adjusted R^2^ = 0.285 (F [5102] = 8.94, *p* < 0.001). Statistical significance was defined as *p* < 0.05.						
(**b**)						
**Predictor**	**β (Coef.)**	**SE**	**z**	***p***	**OR (Exp(β))**	**95% CI for OR**
25(OH) Vitamin D (ng/mL)	−0.042	0.018	−2.33	0.020 *	0.96	0.93–0.99
Weight (kg)	−0.067	0.015	−4.47	<0.001 *	0.93	0.90–0.96
Lean mass (kg)	−0.031	0.011	−2.82	0.005 *	0.97	0.95–0.99
Age (years)	0.040	0.013	3.08	0.002 *	1.04	1.01–1.07
Age at menopause (years)	−0.025	0.014	−1.78	0.075	0.98	0.95–1.00
Constant	3.82	1.23	—	0.001 *	—	—
* Values represent regression coefficients (β), standard errors (SE), Wald z statistics, and corresponding odds ratios (OR) with 95% confidence intervals (CI). Low BMD defined as ≤1 SD below the sample mean (<0.85 g/cm^2^). Model adjusted for serum 25-hydroxyvitamin D [25(OH)D], body weight, lean mass, age, and age at menopause. Model fit: Nagelkerke R^2^ = 0.36; Hosmer–Lemeshow *p* = 0.42. Statistical significance was defined as *p* < 0.05.						

## Data Availability

The datasets presented in this study are not readily available because they are part of an ongoing randomized controlled trial (NCT06741956). Data will be made publicly available upon completion of the trial through the institutional repository of the University of Seville (IDUS). Requests to access the datasets should be directed to the corresponding author.

## Abbreviations

The following abbreviations are used in this manuscript:

25(OH)D	25-hydroxyvitamin D
BMD	Bone Mineral Density
aBMD	Areal Bone Mineral Density
vBMD	Volumetric Bone Mineral Density
DXA	Dual-Energy X-ray Absorptiometry
QCT	Quantitative Computed Tomography
BMI	Body Mass Index
OR	Odds Ratio
